# Analyzing User-Generated Web-Based Posts of Adolescents’ Emotional, Behavioral, and Symptom Responses to Beliefs About Depression: Qualitative Thematic Analysis

**DOI:** 10.2196/37289

**Published:** 2023-01-24

**Authors:** Kim Kristoffer Dysthe, Jan Ivar Røssberg, Petter Bae Brandtzaeg, Marita Skjuve, Ole Rikard Haavet, Asbjørn Følstad, Atle Klovning

**Affiliations:** 1 Department of General Practice/Family Medicine University of Oslo Oslo Norway; 2 Division of Psychiatric Treatment Research, Department of Psychiatry University of Oslo Oslo Norway; 3 Department of Media and Communication University of Oslo Oslo Norway; 4 SINTEF Digital, Sustainable Communication Technologies, Oslo, Norway Oslo Norway

**Keywords:** adolescent, depression, internet, education, preventive psychiatry, early medical intervention, health literacy, cognitive behavioral therapy

## Abstract

**Background:**

Depression is common during adolescence. Early intervention can prevent it from developing into more progressive mental disorders. Combining information technology and clinical psychoeducation is a promising way to intervene at an earlier stage. However, data-driven research on the cognitive response to health information targeting adolescents with symptoms of depression is lacking.

**Objective:**

This study aimed to fill this knowledge gap through a new understanding of adolescents’ cognitive response to health information about depression. This knowledge can help to develop population-specific information technology, such as chatbots, in addition to clinical therapeutic tools for use in general practice.

**Methods:**

The data set consists of 1870 depression-related questions posted by adolescents on a public web-based information service. Most of the posts contain descriptions of events that lead to depression. On a sample of 100 posts, we conducted a qualitative thematic analysis based on cognitive behavioral theory investigating behavioral, emotional, and symptom responses to beliefs associated with depression.

**Results:**

Results were organized into four themes. (1) Hopelessness, appearing as a set of negative beliefs about the future, possibly results from erroneous beliefs about the causal link between risk factors and the course of depression. We found beliefs about establishing a sturdy therapy alliance as a responsibility resting on the patient. (2) Therapy hesitancy seemed to be associated with negative beliefs about therapy prognosis and doubts about confidentiality. (3) Social shame appeared as a consequence of impaired daily function when the cause is not acknowledged. (4) Failing to attain social interaction appeared to be associated with a negative symptom response. In contrast, actively obtaining social support reduces symptoms and suicidal thoughts.

**Conclusions:**

These results could be used to meet the clinical aims stated by earlier psychoeducation development, such as instilling hope through direct reattribution of beliefs about the future; challenging causal attributions, thereby lowering therapy hesitancy; reducing shame through the mechanisms of externalization by providing a tentative diagnosis despite the risk of stigmatizing; and providing initial symptom relief by giving advice on how to open up and reveal themselves to friends and family and balance the message of self-management to fit coping capabilities. An active counseling style advises the patient to approach the social environment, demonstrating an attitude toward self-action.

## Introduction

### Background

Depression is common among adolescents [1-10]. However, adolescents are hesitant to talk openly about their mental health and appear difficult to motivate for therapy owing to stigma and social shame [11-17]. For detecting and treating adolescent depression, current health services have severe disparities, and general practitioners are left with few alternatives for intervention [18,19]. When adolescents are referred to specialist clinics, conditions tend to be more severe [20]. This is unfortunate, given the benefits of early intervention. Depression during adolescence increases the risk of various conditions at a later stage in life, such as mood disorders, anxiety, early disability, suicide, and even severe mental disorders [4,21-25].

With an increased burden on primary health care systems and specialist psychiatric clinics, there is an increased need for alternative ways to intervene [15,19,26]. Previous research suggests giving general practitioners a better vigilance of the signs of depression. At the same time, previous literature suggests implementing easy-to-use means to intervene at an adolescent age, potentially easing the strain on the entire mental health care system [18]. One way to achieve this is to develop targeted psychoeducation (PE) with specific clinical aims and brief cognitive behavioral therapeutic interventions targeting specific traits of adolescent depression [19]. A second path is to engage in innovative services such as supervised peer-to-peer web-based group therapies, automated agents for relevant health information [27], or the use of conversational artificial intelligence (AI) agents (chatbots) for psychoeducational purposes [18]. Such services could meet young peoples’ demand for new ways of conducting therapy with less direct human-to-human contact [28]. Providing atomized PE in ways that require less use of human resources could be something the health care system will be dependent on in the future. In general, the use of technology could be beneficial in the way therapy is conducted and to use health care resources in a more efficient manner.

However, for clinical and technological use, we need PE content that is not only relevant [29-31] but also provides health information with specific intentional responses: to instill hope and thereby reduce therapy hesitancy [32], ease social shame and blame [33], and provide initial symptom relief [33-35]. Previous literature concerning PE suggests that these 3 aims are central to treat adolescents with symptoms of depression [29-36].

### Prior Work

Although we know from earlier research that PE and depression courses may ease depression symptoms in adolescents [37,38], we believe that the full potential of PE is not met until it is derived from data about information needs [31] and responsiveness. In general, PE could be provided through courses and educational programs, technological platforms, or as an integral part of clinical therapies to improve mental health literacy [39]. Traditionally, the informational content of PE is developed from established theories [29,37]. Previous research has addressed the clinical effect of different approaches [37], rather than using empirical data for the development of psychoeducational content [30,33,36,40,41]. Only a few previous studies have derived such content from data-driven analyses [29-31], asking what information is relevant to the target population. We could find no previous research that aimed to investigate the response to PE: What type of health information has the potential to alter emotions, behavior, and symptoms?

This gap in the current research is surprising, considering the abundance of traces of such responses available in web-based environment for adolescent health support. For example, open web-based support services for youth represent rich potential sources of adolescents’ responses to web-based health information, where developers of PE for therapeutic interventions, courses, programs, and information technology (IT) for health support could gather relevant insights into the lifeworld of young people [31].

**Figure 1:**
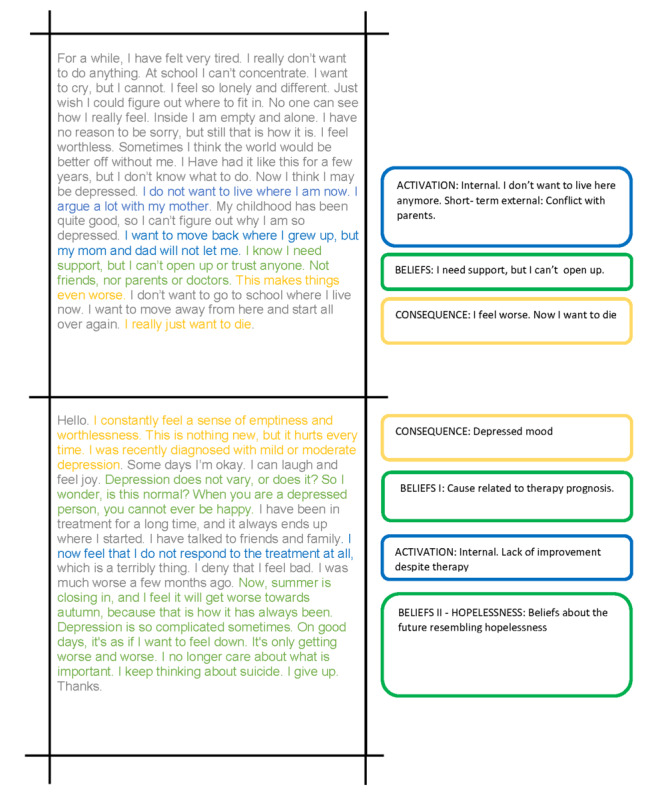
Examples of posts displaying the interpretation of text narratives demonstrating the ABC (activation, beliefs, and consequences) framework (owing to privacy concerns, different posts are paraphrased and altered before being translated to English).

Different depression courses are based on therapeutic models such as interpersonal therapy [34] and cognitive behavioral theory (CBT) [35,42]. In brief, CBT postulates that different negative automatic thoughts (NATs) [35,42] are triggered by activating external or internal events, with an ensuing emotional, behavioral, and symptomatic response. This chain of events is described within an ABC framework [43,44], where the letter A (activation) represents the activating event; B (beliefs) represents the ensuing NAT; and C (consequence) represents the emotional, behavioral, and symptom responses. In short, the basic principles of CBT are based on cognitive restructuring—challenging the logical coherence and veracity of the NAT (B) to generate a preferred response (C). For instance, a responsive NAT about depression as an inherent feature of the personality rather than a disease that can be treated could trigger an emotional response of sadness and even shame, in addition to a set of secondary NATs about the future. In general, the NATs circle around the internal self-concept and its relation to other people as well as the future. In cognitive theory, this is known as Cognitive triad of depression [43]. Figure 1 provides further examples of the ABC framework, NAT, and ensuing responses.

Importantly, cognitive theory distinguishes between underlying core beliefs and cognitive schemas rooted in childhood experiences and genetic precursors as blueprints for NATs. NATs are activated by stressors and, to a lesser degree, present during a euthymic state. However, earlier literature suggests that this distinction is challenging to make during ongoing depression [45]. Therefore, in this study, we used phrase *beliefs* as synonymous to NAT and core beliefs.

### Three Clinical Aims

The 3 clinical aims of instilling hope, reducing shame and guilt, and providing initial symptom relief are nested in prior PE development and research [29-36,46]. These PE programs commonly present different topics of information, that is, information about self-management, treatment options and prognosis, etiology, diagnosis, and natural course. Research also suggests that different emotional and cognitive states are associated with the clinical goals [33,36]:

1. *Instill hope* and thereby reduce *therapy hesitancy* [32].
2. *Reduce shame* through mechanisms of externalization—what the person has is a disorder that can be treated [32,33].
3. *Provide initial symptom relief* by stimulating self-management [35] and social support [33-35].

### Aim of This Study

To meet our clinical aims, we need to know more about the cognitive responses to common beliefs associated with depression, leaving the following research question:

What is the emotional, behavioral, and symptom response following common beliefs about depression found in posts written by adolescents with symptoms of depression asking questions on a web-based service?

Using a rich self-reporting textual data set from a web-based information service, this study aims to be the first to derive population-targeted PE information content with a potential cognitive response effect. The overarching aim is to propose information to support or challenge such beliefs, and thereby develop a population-specific PE for clinical use, in programs or courses or artificially intelligent conversational agents using natural language dialogues (conversational AIs, chatbots). For such PE to meet the clinical aims, we need to know what NATs concerning depression are found possibly responsive. The principal aim of this study was to gain knowledge about the emotional, behavioral, and symptom response to beliefs associated with depression in adolescents.

## Methods

### Research Design

Using a qualitative design, this study uses the advantages of user-generated posts sampled from the main public web-based information service for adolescents and young adults in Norway [47]. The thematic analysis [48] was partly a top-down deductive approach based on PE information topics [24,32,33,36] and partly inductive in nature, generating themes according to the specific findings.

### Data Source

The web-based information site [47] serves as a question-and-answer service about a range of topics relevant to young people, such as contraception, school and education, family relations, work, and mental health. Adolescents using the service (hereafter: the users) can choose the topics *mental health and emotions* from a drop-down menu of 62 main categories. An editorial staff of physicians, psychologists, and nurses answered the questions. Users can type up to 1000 signs per post. The service allowed all the participants included in our sample to specify gender as female, male, or others, and age as aged from 13 to 20 years, currently with the additional alternatives of *below 13* and *above 20*. Both the questions posted and the replies from the editorial staff were published on the site. In a few posts, a request for privacy was stated. Consequently, all the posts were anonymized upon delivery by permanently deleting the IP addresses.

On replying, the editorial staff labeled the posts into subcategories, one of which is *depression*. This assessment is made either by recognizing the symptoms of depression, as stated in the posts, or by interpreting the entire post as displaying a depressed mood.

Interestingly, to explain the background of the question, the users tend to describe a sequence of events leading to a mental state, thus revealing general knowledge, insufficient knowledge (cognitive deficits), or misconceptions (cognitive distortions) about the condition [36]. In addition, they portrayed small narratives displaying emotional, behavioral, and symptom responses to different beliefs and activating factors. As displayed in Figure 1, most of the posts are short but rich in content.

Although most studies in this field rely on structured data from questionnaire-based surveys, the data presented herein are spontaneous and unstructured. Hence, our data represent a highly relevant and unique way to reveal not only how adolescents experience symptoms of depression but also how they express such experiences in written words, what message they automatically choose to convey to receive a relevant reply, and how they choose to convey the narratives leading to the current state. This is illustrated in Figure 1. In this manner, self-reporting data serve as an important contribution to the research in this field [49-51]. In addition, previous research suggests that self-perceived mental states correspond well with the clinical diagnoses given after a professional assessment [52-54].

### Data Sampling

In total, the data set consisted of 256,000 user-generated posts comprising all the different topics delivered in Microsoft Excel (Microsoft Corp) format through a data delivery agreement between the project and The Norwegian Directorate for family, adolescence, and child affairs. Each post was assigned a unique ID number. The main categories, subcategories, year, age, and gender serve as Excel column headlines. Owing to privacy issues, as stated in the data delivery agreement, we avoid quoting the posts verbatim. Instead, we made minor changes to the syntax and wording of the extracts quoted in this report, still conserving the content of meaning. This diminishes the possibility of backtracking the extracts through web-based translation and search engines to the original post published on the web-based information site [47].

The data sampled for this study consisted of posts written by adolescents aged 16-20 years. The depression subcategory included 870 posts. For a previous content analysis [31], we randomly sampled 100 posts to provide the required statistical strength. We simultaneously conducted the analysis for this study, and no new codes appeared after 34 posts. In this 100-post sample, 71% (71/100) were posted in the years 2015 to 2018, with the highest proportion posted in 2017 (33 posts). Only 12% (12/100) the posts were submitted from 2009 to 2012. Of the 100 posts, 75 (75%) were written by female individuals, 22 (22%) by male individuals, and 3 (3%) stated gender as *other*. The sample age was quite evenly distributed.

### Ethical Approval

The study has been reviewed by the Regional Research Ethics Committee. Based on the application, the project does not qualify as medical or health research and is therefore outside the scope of the Health Research Act in Norway (as per §2). This is because the data material is anonymized. The decision has reference number 68312.

### Data Analysis

The 2 raters who conducted the analysis (KKD, a general practitioner, and JIR, a psychiatrist) were both clinically trained and educated in CBT [31,49-54].

Both raters followed the thematic analytical process described by Braun and Clarke [48]. The analysis was partly top-down deductive based on PE information topics [24,32,33,36] and partly inductively generating themes by interpreting the responsive cognitive consequences revealed in the posts. Themes were constructed from the initial coding using a 5-step process:

1. Step 1: The 2 raters started out by familiarizing themselves with the data, reading the posts closely. Both raters identified posts describing symptoms of depression (66/100) and different beliefs about depression and recognized the ABC frameworks in the posts’ narratives. These posts (53/100, 53%) were marked for further analysis.
2. Step 2: The first rater (KKD) coded the posts using the NVivo Pro (version 12; QSR International) application for qualitative analysis. Both raters identified 2 levels of codes, following Beck’s cognitive theory [43]: beliefs about depression (B) were coded according to the psychoeducational information topics. Six codes were identified: beliefs about etiology, natural course, therapy, diagnosis, rights, and self-management including social support. Descriptions of emotional states, behaviors, and symptom alterations displaying the different responses (C) within the ABC framework were then coded. The second rater (JIR) read the posts and agreed with or disapproved the initial coding. After 10, 40, 80, and 100 posts, the coders met to discuss. Overall, 22% (22/100) of the total number of text references revealed an initial divergence and a subsequent agreement. After agreeing about the initial coding or new codes suggested by both raters, only 1 of the text references remained unresolved.
3. Step 3: KKD sorted these codes into themes displaying the emotional, behavioral, and symptom responses following beliefs (B) about depression. KKD then derived the subthemes according to the different types of beliefs (B) before the ensuing responses (C).
4. Step 4: During a themes review process, KKD read the posts several times to review the narrative structures of the posts and to ensure as little overlap between themes as possible.
5. Step 5: The most frequent themes were presented to the entire group of authors to conduct a finishing theme review and give each theme and subtheme their final headings.

## Results

### Study Characteristics

The thematic analysis yielded 4 themes displaying the different emotional, behavioral, and symptom responses and 11 subthemes reflecting the different psychoeducational information topics. Themes and subthemes are presented in Table 1.

**Table 1:** Themes and subthemes; common primary beliefs (B1); secondary beliefs (B2); and the ensuing emotional, behavioral, and symptom responses (C1 and C2).

Themes and subthemes	Emotional, behavioral, and symptom responses
1. Hopelessness Etiology and causal attributions Mood variation and natural course Therapy alliance and availability	B: Cause determining resilience, therapy options, and prognosis. C: Depressed mood associated with B2: present and past states are stable. B: Beliefs about the condition as stable, relentlessly deteriorating. C: Depressed mood associated with B2: beliefs about present and past states being stable. B: Thought about the inability to relate to a therapist, and therapy being unavailable. C: Depressed mood associated with B2: the present and past states are stable.
2. Therapy hesitancy Therapy prognosis Confidentiality and rights	B: It did not help before: it will not help again. C1: Depressed mood associated with—B2: beliefs about present and past states being stable and will continue—C2: Resulting in therapy hesitancy. B: The therapist will tell my parents everything. Depression will never change. The only hope is documentation to unleash rights. C: Therapy hesitancy.
3. Shame and stigma Give a diagnosis	B: I cannot cope, but I do not know why. C: Reduced daily function and a vague sensation of mental illness associated with the emotional state of shame.
4. Symptom response Self-management Opening to friends or family Inherent social dysfunction Impose a burden on others Attitude of self-action	B: Other people perform perfectly well. Due to my depression, I am unable to cope. B: Opening to friends and family will make me feel better, but I don’t know how to open. B: I am not able to establish or sustain social relations. B: By opening I will impose a burden on friends or family. C: More symptoms when experiencing little social support. B: There are things I have done to cope. I try to obtain social support. C: Experiencing less symptoms and suicidal thoughts.

### Theme 1: Hopelessness

#### Overview

A principal finding of this theme is that hopelessness appears as a cognitive construction of beliefs stemming from different cognitive styles and is affected by a depressed mood, as listed in Table 2. We identified posts describing hopelessness that conveyed a set of beliefs about the future originating from a negative self-concept and worthlessness in association with others. Constituting the subthemes of causal attribution, natural course, and prognosis, this indicates beliefs about the state of depression as something anchored in unalterable life narrative less prone to therapeutic intervention or natural fluctuations. Supporting the apprehension of hopelessness as a mood-moderated set of beliefs, the state of hopelessness appears from an A-B1-C-B2 framework, whereas B1 performs as primary beliefs about depression described in subthemes 1 to 3, C is the resulting mood, and B2 is a set of different beliefs about future events affected by a depressed mood, as displayed in Table 1.

We found 3 classes of B1 beliefs that lead to the cognitive state of hopelessness: etiology and causal attributions, mood variation and natural cause, therapy option and alliance.

**Table 2:** Beliefs conveying a message of hopelessness and different cognitive styles.

Rephrased extracts resembling hopelessness	Interpretation	Cognitive style
“I no longer think I will stay healthy in the future” [ID_214457]	Do not know what the future will bring. Sees no way out of a troublesome situation	On the basis of assumptions that the present state condition is stable and will continue
“it just repeats itself again and again” [ID_204202]	The future will look like the past	On the basis of assumptions that the past state condition is stable and will continue
“I know the only one that can help me is myself, but I really can’t cope anymore....please, give me something to hope for.” [ID_143601]	I feel unable to manage; I give up	The situation is stable, based on negative, global self-characteristics
“Really don’t know what to do and even why I am alive” [ID_96287] “I just want to die. I have tried everything” [ID_258080]	No real options left. Bewildered. I want to die. Thoughts about dying as a possible solution	On the basis of assumptions that the present state condition is stable and that there is no way out

#### Etiology and Causal Attributions

Referring to beliefs about etiology and causal attributions, we found beliefs in the user-generated posts about a direct causal link between stressors, risk factors and depression to determine intervention options. First, a few posts portrayed beliefs about life experiences weakening social competence. Second, we found descriptions of causal attributions about a direct causality between stressors, risk factors, and the condition, implying that therapeutic or self-management interventions can only target the cause and that this presumed cause determines if it is within therapeutic reach and what type of intervention it is subject to. Third, long-term depression can make one attuned with the symptoms, implying that depression enters a habitual state:

I’ve had a secure and mostly uncomplicated childhood, so I don’t know why I feel this way. Should I seek professional help, or manage on my own?

ID_113789

Only at times my childhood has been slightly troublesome, still I keep pushing people away from me.

ID_204202

I have read online that depression can be caused by adverse events...That is why I wonder why I’m depressed and what I can do to get better.

ID_98556

I’ve felt depressed for such a long time that being happy feels uncomfortable.

ID_119794

#### Mood Variation and Natural Course

Some of the contributors viewed depression as a condition that relentlessly developed into a more serious state. Depression seems to be viewed as a state from which there is no escape, leaving a sense of bewilderedness, in addition to worries about acting on suicidal thoughts in a somewhat deterministic manner:

Everything just goes downhill, why do I live? It has been like this for periods during my teen years, but the last year has been much worse. ...To be honest, I don’t see light at the end of the tunnel. ...This may sound gloomy, but that’s how I feel, and how I have felt for a while.

ID_240835

#### Therapy Options and Alliance

Thoughts about therapy seem to trigger an ensuing state of hopelessness in 3 different ways. First, the posts reveal a lack of belief in the ability to open up to a therapist, imposing it as a burden on themselves to establish a sturdy alliance. The contributors seemed reluctant to share and be open about mental problems with friends, family, and a therapist. Even when realizing a need for help, it seems that the alliance must be built on trust and provide the experience of being taken seriously:

I know I need help, but I can’t open or trust anyone! Not friends, nor parents or doctors.

ID_114994

I’ve been seeing a psychologist but can’t open up to him...he just tells me things I already know; no therapist will ever take me seriously.

ID_84402

Second, the posts describing beliefs about availability also display a perception of therapy as the only, final solution. Finally, beliefs about hospitalization and therapy prognosis seemed to stem from previous experience with hospital treatment, nested in the conception that what happened before will happen again:

They will not take me seriously and send me somewhere else.

ID_84402

Being admitted to hospital just feels like being stored away.

ID_157360

After seeing a psychologist, I just told my mother I was better so that I didn’t have to go there anymore. The treatment did not help anyway...

ID_119794

### Theme 2: Therapy Hesitancy

#### Overview

The responses (C) following thoughts about depression constituting theme 2 are not an emotional state but rather a description of hesitancy to seek professional help. We found 2 classes of beliefs triggering this type of therapy-related behavior: therapy prognosis and confidentiality and rights.

#### Therapy Prognosis

Following the A-B1-C-B2 framework of theme 1, we found an interplay between themes 1 and 2 in a few posts describing negative thoughts about the effect of hospital treatment and therapy prognosis. It appears that a secondary consequence (C2) follows the state of hopelessness (B2): a behavioral response (C2) of therapy hesitancy displaying an A-B1-C1-B2-C2 framework.

#### Confidentiality and Rights

One post described concern about confidentiality as a key issue when considering therapy, revealing a lack of knowledge about the current legislation:

If I talk about my thoughts and problems to the nurse, must she tell mom or anyone else?

ID_120532

A few posts revealed a demand for knowledge about rights concerning absence from school or work when having depression, asking whether professional documentation could help. Such beliefs were associated with beliefs about depression as a steadfast condition related to the subthemes of natural course and therapy prognosis. However, these posts portrayed no state of hopelessness, probably because of a hope that documentation can unleash rights, making work or education possible despite an ongoing depression*.*

### Theme 3: Shame and Stigma

A proportion of the posts described a somewhat vague sensation that something was mentally wrong, leaving an experience of shame concerning social, familial, and school dysfunction. The consequence of the ensuing emotional state of shame appears to be preceded by the experience of dysfunction. The sited post described a demand for knowing what the symptoms may represent, interpreted as a possible consolation:

My friends do things every day and always ask if I want to join. I really like them, but I...always try to come up with excuses to avoid going out. ...I have lost motivation for school. It is extremely difficult to concentrate. ...I wonder what these things represent. Depression?

ID_245191

### Theme 4: Symptom Response

#### Overview

Alterations in symptoms, as described in the posts, seem to be related to beliefs about self-managing options and the ability to initiate and sustain social participation and support. In general, negative responses of experiencing little or no social support when depressed have been described in a large number of posts. In addition, the posts portray the ensuing experiences that model existing beliefs and offer new or enhanced expectations. The theme describes different beliefs about social interaction and the ensuing negative symptom responses. Finally, some of the posts described both social interaction and other measures of self-management to cause a symptoms respite or reduced suicidal ideation.

Symptom response is described within this framework as the depressive symptomatic state before activating beliefs and descriptions of how such beliefs give rise to additional symptoms or symptom relief.

#### Self-management

A few posts addressed different self-management measures. Some of these posts resemble theme 3 in that they also describe an experience of relative dysfunction followed by a sensation of shame; however, this is related to an expectation of coping despite an ongoing known condition. In addition, beliefs that one should be able to manage like all others do imply a false insight into people’s minds and coping abilities (ID_258080). One post suggests traveling to another country as the only possible solution, serving as an example of how unrealistic hopes and plans may affect mood:

I will travel abroad for a while just think about something else. Now I feel life is a prison.

ID_240835

#### Opening to Friends or Family

Related to theme 3, one post suggested informing friends about the condition through social media, expecting this to give relief. More generally, the posts demanded information about how to open up to friends and family as a possible road map for mood improvement. Some posts also described the challenges in sharing difficult experiences with parents or friends, experiencing a sense of loneliness and worsening symptoms:

I can’t open about this to my parents. I feel so alone. I don’t talk to them because they will only judge me.

ID_185571

#### Inherent Social Dysfunction

Beliefs about inherent social dysfunctions seemed to hamper the ability to open up to friends and family. This, in contrast to the theme 3 posts, does not display the emotion of shame. We could also identify beliefs and experiences regarding how a mental or physical condition will make you lose friends and support. The posts described being left alone with negative thought patterns and the corresponding negative symptom response as something placing an extra burden on the situation*.* In addition, experiencing social interactions as a challenge seems to enhance such beliefs. Thus, avoiding troubling social relationships results in loneliness, eventually leading to symptom deterioration:

I’m not able to retain social contact, I think I’ve lost all my friends. I would like to see my friends more, but now I feel exhausted...

ID_196669

#### Impose Burden on Others

The posts constituting this subtheme described a hesitancy to open up to family or friends based on the assumption that seeking support will impose a burden on them, thereby hampering disclosure. One post described talking to friends about suicidal thoughts as something that could eventually exhaust them. However, hiding such thoughts made the relationship less challenging, improving social interaction and support, even if it meant keeping suicidal thoughts to oneself:

I keep my negative thoughts to myself, so I don’t put too much strain on them. Now we don’t argue anymore, so it works, actually. Psychologist didn’t help me though.

ID_262215

#### Attitude of Self-action

We found only a few posts describing direct actions to relieve symptoms of depression. One post described how asking advice from others made the contributor positive to seek professional help, leaving a hope for improvement. Two other posts described support from parents and siblings to reduce suicidal ideation:

The only thing that prevents me from committing suicide is that I can’t bear the thought of what it would do to my family.

ID_119794

A single post described physical exercise to achieve improvement. One post described hiking to relieve symptoms. Another post suggested sharing the diagnosis on social media: if friends knew about the diagnosis, it would come as a relief, and possibly make them more understanding and supportive*.*

## Discussion

### Principal Findings

As indicated in the first clinical aim of PE, conquering hopelessness seems essential to reduce therapy hesitancy and to improve therapy motivation and adherence [32].

In addition, a growing body of research links hopelessness to suicidal ideation [55]. This concept has received increasing clinical attention. The field of cognitive psychology views hopelessness as a set of negative thoughts about the future, constituting one of the three corners of the cognitive triad of depression, in addition to Aaron Beck’s theory of hopelessness and its clinical application—Beck’s Hopelessness Scale [56]. The cognitive depression triad of Beck assumes a distorted expectancy toward future events leading to depression, whereas similar views about the future, as described in the theory of hopelessness, result *from* a depressed mood.

From the results of this study, we suggest 2 ways to challenge hopelessness. First, previous literature classifies hopelessness in different ways. Although some studies originally defined it as a secondary emotion [57], the schism between affection, mood, emotional, and cognitive states is blurry [57,58]. If we interpret hopelessness as a set of secondary beliefs (B2), we could possibly challenge beliefs directly through CBT interventions before routing therapists through the entire ABC framework. Therefore, we recommend a direct clinical approach targeting the state of hopelessness.

Second, theme 1 analysis also implies targeting (B1) beliefs as displayed in the different subthemes, targeting hopelessness indirectly by means of the ABC framework by also challenging beliefs about causal attributions and natural course. These approaches need to be nuanced; the state of hopelessness could at times stem from arguably negative life events, difficult societal conditions, and the perception of negative trends in world development. From a clinical perspective, acknowledging such beliefs may be a chief ingredient to maintain alliance. Consequently, cognitive reattribution should focus on erroneous beliefs about depression and beliefs related to Beck’s triad, that is, beliefs concerning the internal self-concept.

Beliefs about therapy alliance found in a few posts of the *therapy alliance and availability* subtheme revealed that some of the contributors were reluctant about visiting a physician, assuming they were unable to open to a professional helper. However, previous studies have not addressed this issue. As such, beliefs about establishing an alliance may depend on patients’ abilities and skills. As an information topic to stimulate help seeking, counselors should consider talking openly about the role of the therapist and the working alliance when initiating a clinical encounter with adolescents. We could also provide such knowledge through IT such as chatbots. Our results also point at the chief beneficial aspects of building a strong alliance. As adolescents do not visit their physician frequently, our findings suggest a special awareness about initiating a sturdy physician-patient relationship in this age group as a prerequisite for later help seeking.

Two factors seem important regarding the theme 2 issue of *therapy hesitancy*. First, in the mixed pattern of secondary beliefs (B2) about hopelessness and therapy hesitancy, we might have observed possible mechanisms behind the postulated and observed response of help seeking and therapy adherence from information intended to instill hope [32,46]. The secondary beliefs (B2) resembling hopelessness coincide with an intention or behavior of not seeing a physician or continuing therapy. By turning this the opposite way, information challenging erroneous primary beliefs (B1) about therapy prognosis and hospital treatment could forestall the desired theme 3 positive response of therapy attendance through the cognitive state of hope.

Second, challenging therapy hesitancy is not only a question of informing about issues such as confidentiality legislation, but also discuss parent involvement and a reasonable degree of nondisclosure to establish or preserve therapy alliance. This issue should also nourish the debate on free health services throughout adolescence.

About theme 3 “Shame and stigma,” we focus on the mechanism of externalization through the emotion of social shame, activated by a sensation of underachievement [59]. From the results of this study, as a clinical and informational approach to diminish shame, we should perhaps be less reluctant to diagnose depression despite concerns about stigmatization, as portrayed by previous research [11]. Second, back to the theme 2—in general, the feeling of shame seemed to fetter the ability to be open about the condition. When previous research links shame and stigma to therapy hesitancy, health information provided by technologies such as chatbots or through PE programs or courses should be clear about addressing symptoms as signs of a possible disease, despite the risk of stigmatization. This finding may also imply that clinicians are less reluctant to provide a tentative diagnosis.

With regard to theme 5, we found positive and negative symptom responses following beliefs chiefly related to self-management measures, social support, and participation. Interestingly, this resonates with previous research indicating a demand for information about self-management among adolescents who are depressed [29-31]. This study also provides additional insights into the content of targeted clinical and psychoeducational advice to stimulate social interaction and skills while engaging in pleasant activities.

Recent studies on psychotherapeutic measures have increasingly focused on the possible negative side effects of psychological interventions [16,31,37]. In this regard, we may interpret the subtheme of *self-management* with caution. Stimulation of self-management has long been the chief goal of PE development and clinical therapy. Although only a few posts revealed unrealistic beliefs about coping abilities, this indicates that such beliefs are present. Therefore, a thorough examination of self-assessed coping abilities should receive clinical attention.

Previous studies have indicated social support as an important component of coping [29-31,34,60]. This study also revealed beliefs about social support as challenging to establish or sustain.

Interestingly, we interpreted positive experiences in the subtheme of “Attitude of self-action” when the writer described a more direct approach asking friends for concrete advice, revealing the need for social support. Although such descriptions were outnumbered by descriptions of various forms of dysfunction, qualitatively they may propose clinical advice to conquer assumptions about a stable, fixed social environment. Instead of recommending social support as something that is received from the environment, the therapist may emphasize a more direct approach, implying an active counseling style that guides the patient to consult the social network with an attitude of seeking advice about self-actions.

### Implications for Future Work

Although help-seeking behavior and therapy motivation could be more challenging to measure, we could design studies to investigate the effect of targeted, relevant information on symptom responses. In this way, future research could investigate the efficacy of chatbot prototypes providing relevant and responsive PE, eventually using technology for symptom detection in textual data [61]. If specific information about depression affects mood by influencing common beliefs in adolescents who are depressed, research on the same textual data could look further into *ways of thinking (cognitive distortions)* [62], which might be characteristic of an adolescent brain undergoing depression. We also suggest further research on the response to psychoeducational and clinical guidance about self-management and possibly reconsider giving the topic such a dominant role in PE courses or technology-based information solutions. In general, we believe that future studies on therapeutic measures, both face-to-face and IT interventions, should investigate possible side effects of health information and CBT targeting the adolescent population.

Investigating if what we expect from the cognitive functions of the adolescent brain corresponds with specific empirical findings, developing a clinical approach to challenge such ways of thinking may be a way to develop therapeutic tools for clinical and technological use. The existing conversational agent Woebot is an example of a conversational AI providing information and mental exercises, making the user conscious about dysfunctional ways of thinking and the association with depression. Overall, by combining population-specific PE with therapeutic tools to challenge common cognitive distortions, we could build a brief CBT toolkit that is prone to further examination, for instance, using a clinical trial design. By applying the same methods of analysis on similar web-based textual data, we could also develop technologies and information aiming at other populations and different mental diseases. Furthermore, designing joint technological and clinical solutions may be a way to meet the growing demand for more specialized approaches targeting adolescent mental health.

Although the aim of this study is to provide both psychoeducational content for technological use and to give the therapist a head start stimulating therapy motivation and offer initial symptom relief, this novel way of creating therapy tools from empirical data is also applicable to other fields using similar data from different sources. This could be done using clinical interviews supplementing self-reporting data for the same type of empirical development. Previous research has showed that adolescents appreciate support from conversational agents such as Woebot [63]. However, the field of adolescent mental health is complex and involves different life areas such as school, social life, parents, and family. Future work should focus on creating technologies and therapeutic tools to coexist with different types of joint face-to-face and technological therapies.

### Limitations

As both raters possess CBT education and training, analyzing the posts with reference to beliefs associated with symptoms could easily make us interpret every belief or statement as something prone to cognitive restructuring, expecting an ensuing symptom relief. Anchoring the analysis to CBT could also make us biased toward considering such responses as more generally inherent than what is real, thus overinterpreting the themes to possess an exaggerated clinical significance.

To compensate for this, we made the following reflections: first, during coding, we openly discussed different possible interpretations regarding language, comorbid conditions, and challenges analyzing the different patterns of the ABC framework. Second, both raters could effortlessly connote the posts to real patient histories. Adding to the theoretical anchoring and the concurrent reflection on possible biased interpretations, we used our therapeutic experience to tie the analysis to real-world clinical situations. In addition, the team included 2 other general practitioners with CBT education and training. During theme construction, the 2 raters (KKD and JIR) and the entire team met for a final theme review session to discuss interpretation related to theory, therapeutic experience, and clinical implications.

We acknowledge that not every adolescent presents the beliefs and the cognitive structure of responses found in this study. Rather, those experiencing depressive symptoms in a certain life context could end up posting a question on a web-based public service. Despite this obvious selection bias, we believe that this type of data could provide supplementary insights. Regarding transferability, knowledge like this could come to use the moment we discover depressive symptoms in an individual belonging to the current population, thus providing a prior understanding of adolescents’ response to different beliefs associated with depression.

Moreover, when a stressor was not apparent in the posts, some of the beliefs appeared as generalized, resembling core beliefs or rules, as described in the Introduction section. However, we did not consider this limitation to weaken the applicability of this study, although we viewed core beliefs as more resistant to cognitive restructuring. Regardless of the classification, all levels of beliefs should be challenged during CBT interventions.

Finally, using the advantages of self-reporting data, we were unable to control data collection, making the analysis vulnerable to confounding factors such as common comorbid psychiatric conditions, drug abuse, and random mood swings. Although the posts were collected from as far back as 2009, many of them were submitted between 2013 and 2018, raising 2 main concerns.

First, changes in language or language differences among socioeconomic classes could influence words and phrases interpreted as depressive symptoms. To address this, we looked for beliefs about depression anchored in previous theories directing language interpretation.

Second, we acknowledge that major events have taken place after 2018. The global COVID-19 pandemic, the emergence of climate change, and the war in Ukraine could possibly provide different results regarding the topic of hopelessness. Obtaining the same type of data after 2019 could gain interesting insights into how young people view future challenges considering the current world situation [64]. However, in this study, we focused on beliefs about depression related to the internal self-concept: how individuals relate to their local social environment, and how the prospect of hopelessness stems from beliefs about the self, rather than being imposed by external stressors such as the climate crisis or the pandemic.

### Conclusions

We advise developers of technological solutions for mental health information and PE courses to give health information that aims to do the following:

1. *Instill hope* and thereby reduce therapy hesitancy by challenging negative beliefs about the future and to explain how such beliefs are affected by depressed mood; explain that there is no causal link between the course of depression and early life experiences, stressors, and chronicity; and inform about the importance of the therapy alliance and that the responsibility of establishing a sturdy alliance rests on the therapist.
2. *Reduce shame* through mechanisms of externalization by providing a tentative diagnosis despite the risk of stigmatizing.
3. *Provide initial symptom relief* by giving advice on how to open to friends and family, and balance the message of self-management to fit coping capabilities. Use an active counseling style advising the patient to approach the social environment, demonstrating an attitude of self-action.

## Abbreviations

AI: artificial intelligence
CBT: cognitive behavioral therapy
NAT: negative automatic thought
PE: psychoeducation

## References

[1] Jane Costello E, Erkanli A, Angold A (2006). Is there an epidemic of child or adolescent depression?. J Child Psychol Psychiatry.

[2] Crockett MA, Martínez V, Jiménez-Molina Á (2020). Subthreshold depression in adolescence: gender differences in prevalence, clinical features, and associated factors. J Affect Disord.

[3] Haavet OR, Dalen I, Straand J (2006). Depressive symptoms in adolescent pupils are heavily influenced by the school they go to. A study of 10th grade pupils in Oslo, Norway. Eur J Public Health.

[4] Kessler RC, Berglund P, Demler O, Jin R, Merikangas KR, Walters EE (2005). Lifetime prevalence and age-of-onset distributions of DSM-IV disorders in the National Comorbidity Survey Replication. Arch Gen Psychiatry.

[5] Kramer T, Garralda ME (1998). Psychiatric disorders in adolescents in primary care. Br J Psychiatry.

[6] Merikangas KR, He J, Burstein M, Swanson SA, Avenevoli S, Cui L, Benjet C, Georgiades K, Swendsen J (2010). Lifetime prevalence of mental disorders in U.S. adolescents: results from the National Comorbidity Survey Replication--Adolescent Supplement (NCS-A). J Am Acad Child Adolesc Psychiatry.

[7] Mojtabai R, Olfson M, Han B (2016). National trends in the prevalence and treatment of depression in adolescents and young adults. Pediatrics.

[8] Rao W, Xu D, Cao X, Wen S, Che W, Ng CH, Ungvari GS, He F, Xiang Y (2019). Prevalence of depressive symptoms in children and adolescents in China: a meta-analysis of observational studies. Psychiatry Res.

[9] Whiteford HA, Degenhardt L, Rehm J, Baxter AJ, Ferrari AJ, Erskine HE, Charlson FJ, Norman RE, Flaxman AD, Johns N, Burstein R, Murray CJ, Vos T (2013). Global burden of disease attributable to mental and substance use disorders: findings from the Global Burden of Disease Study 2010. Lancet.

[10] Wiens K, Williams JV, Lavorato DH, Duffy A, Pringsheim TM, Sajobi TT, Patten SB (2017). Is the prevalence of major depression increasing in the Canadian adolescent population? Assessing trends from 2000 to 2014. J Affect Disord.

[11] Barney LJ, Griffiths KM, Jorm AF, Christensen H (2006). Stigma about depression and its impact on help-seeking intentions. Aust N Z J Psychiatry.

[12] Brandtzaeg PB, Lüders M, Spangenberg J, Rath-Wiggins L, Følstad A (2015). Emerging journalistic verification practices concerning social media. Journalism Pract.

[13] Richardson T, Stallard P, Velleman S (2010). Computerised cognitive behavioural therapy for the prevention and treatment of depression and anxiety in children and adolescents: a systematic review. Clin Child Fam Psychol Rev.

[14] Rickwood DJ, Deane FP, Wilson CJ (2007). When and how do young people seek professional help for mental health problems?. Med J Aust.

[15] Singh SP, Paul M, Ford T, Kramer T, Weaver T, McLaren S, Hovish K, Islam Z, Belling R, White S (2010). Process, outcome and experience of transition from child to adult mental healthcare: multiperspective study. Br J Psychiatry.

[16] Lopez MA, Toprac MG, Crismon ML, Boemer C, Baumgartner J (2005). A psychoeducational program for children with ADHD or depression and their families: results from the CMAP feasibility study. Community Ment Health J.

[17] Lamb C, Murphy M (2013). The divide between child and adult mental health services: points for debate. Br J Psychiatry Suppl.

[18] Roche E, O'Sullivan R, Gunawardena S, Cannon M, Lyne JP (2020). Higher rates of disengagement among young adults attending a general adult community mental health team: time to consider a youth-specific service?. Early Interv Psychiatry.

[19] Iliffe S, Williams G, Fernandez V, Vila M, Kramer T, Gledhill J, Miller L (2009). Treading a fine line: is diagnosing depression in young people just medicalising moodiness?. Br J Gen Pract.

[20] Skurtveit S, Bramness JG, Hjellvik V, Hartz I, Nesvåg R, Hauge LJ, Handal M (2018). Increase in diagnosis of depressive disorders contributes to the increase in antidepressant use in adolescents. Acta Psychiatr Scand.

[21] Fusar-Poli P, Nelson B, Valmaggia L, Yung AR, McGuire PK (2014). Comorbid depressive and anxiety disorders in 509 individuals with an at-risk mental state: impact on psychopathology and transition to psychosis. Schizophr Bull.

[22] Häfner H, Maurer K, Trendler G, an der Heiden W, Schmidt M (2005). The early course of schizophrenia and depression. Eur Arch Psychiatry Clin Neurosci.

[23] Judd LL, Akiskal HS, Paulus MP (1997). The role and clinical significance of subsyndromal depressive symptoms (SSD) in unipolar major depressive disorder. J Affect Disord.

[24] Lewinsohn PM, Rohde P, Seeley JR, Klein DN, Gotlib IH (2000). Natural course of adolescent major depressive disorder in a community sample: predictors of recurrence in young adults. Am J Psychiatry.

[25] Yates P, Kramer T, Garralda E (2004). Depressive symptoms amongst adolescent primary care attenders. Levels and associations. Soc Psychiatry Psychiatr Epidemiol.

[26] Birmaher B, Brent D, Bernet W, Bukstein O, Walter H, Benson RS, Chrisman A, Farchione T, Greenhill L, Hamilton J, Keable H, Kinlan J, Schoettle U, Stock S, Ptakowski KK, Medicus J, AACAP Work Group on Quality Issues (2007). Practice parameter for the assessment and treatment of children and adolescents with depressive disorders. J Am Acad Child Adolesc Psychiatry.

[27] D'Alfonso S, Phillips J, Valentine L, Gleeson J, Alvarez-Jimenez M (2019). Moderated online social therapy: viewpoint on the ethics and design principles of a web-based therapy system. JMIR Ment Health.

[28] Yap MB, Reavley N, Jorm AF (2013). Where would young people seek help for mental disorders and what stops them? Findings from an Australian national survey. J Affect Disord.

[29] Bevan Jones R, Thapar A, Rice F, Beeching H, Cichosz R, Mars B, Smith DJ, Merry S, Stallard P, Jones I, Thapar AK, Simpson SA (2018). A web-based psychoeducational intervention for adolescent depression: design and development of MoodHwb. JMIR Ment Health.

[30] Bru L, Solholm R, Idsoe T (2013). Participants’ experiences of an early cognitive behavioral intervention for adolescents with symptoms of depression. Emotion Behav Difficult.

[31] Dysthe KK, Haavet OR, Røssberg JI, Brandtzaeg PB, Følstad A, Klovning A (2021). Finding relevant psychoeducation content for adolescents experiencing symptoms of depression: content analysis of user-generated online texts. J Med Internet Res.

[32] Nangle D, Hansen D, Grover R, Kingery J, Suveg C (2016). Treating Internalizing Disorders in Children and Adolescents Core Techniques and Strategies.

[33] Fristad M, Goldberg AJ, Leffler J (2011). Psychotherapy for Children with Bipolar and Depressive Disorders.

[34] Mufson L, Dorta K, Moreau D, Weissman M (2011). Interpersonal Psychotherapy for Depressed Adolescents Second Edition.

[35] Clarke G, Lewinsohn PM (2014). The coping with depression course: a group psychoeducational intervention for unipolar depression. Behav change.

[36] Walsh J (2010). Psychoeducation in Mental Health.

[37] Bevan Jones R, Thapar A, Stone Z, Thapar A, Jones I, Smith D, Simpson S (2018). Psychoeducational interventions in adolescent depression: a systematic review. Patient Educ Couns.

[38] Merry S, McDowell H, Hetrick S, Bir J, Muller N (2004). Psychological and/or educational interventions for the prevention of depression in children and adolescents. Cochrane Database Syst Rev.

[39] Jorm AF (2012). Mental health literacy: empowering the community to take action for better mental health. Am Psychol.

[40] Ardito RB, Rabellino D (2011). Therapeutic alliance and outcome of psychotherapy: historical excursus, measurements, and prospects for research. Front Psychol.

[41] Miller W, Rollnick S (2012). Motivational Interviewing Third Edition Helping People Change.

[42] Lewinsohn PM, Clarke GN, Hops H, Andrews J (1990). Cognitive-behavioral treatment for depressed adolescents. Behav Ther.

[43] Beck A, Rush A, Shaw B, Emery G (1987). Cognitive Therapy of Depression.

[44] Ellis A, Dryden W (2007). The Practice of Rational Emotive Behavior Therapy: Second Edition.

[45] Gladstone G, Parker G (2001). Depressogenic cognitive schemas: enduring beliefs or mood state artefacts?. Aust N Z J Psychiatry.

[46] Tursi MF, Baes CV, Camacho FR, Tofoli SM, Juruena MF (2013). Effectiveness of psychoeducation for depression: a systematic review. Aust N Z J Psychiatry.

[47] Ung.no homepage. Ung.no.

[48] Braun V, Clarke V (2012). Thematic analysis. APA Handbook of Research Methods in Psychology, Vol. 2. Research Designs: Quantitative, Qualitative, Neuropsychological, and Biological.

[49] Jackson RG, Patel R, Jayatilleke N, Kolliakou A, Ball M, Gorrell G, Roberts A, Dobson RJ, Stewart R (2017). Natural language processing to extract symptoms of severe mental illness from clinical text: the Clinical Record Interactive Search Comprehensive Data Extraction (CRIS-CODE) project. BMJ Open.

[50] Park S, Lee SW, Kwak J, Cha M, Jeong B (2013). Activities on Facebook reveal the depressive state of users. J Med Internet Res.

[51] Seabrook EM, Kern ML, Fulcher BD, Rickard NS (2018). Predicting depression from language-based emotion dynamics: longitudinal analysis of Facebook and twitter status updates. J Med Internet Res.

[52] Baines T, Wittkowski A (2013). A systematic review of the literature exploring illness perceptions in mental health utilising the self-regulation model. J Clin Psychol Med Settings.

[53] Cohen JR, So FK, Young JF, Hankin BL, Lee BA (2019). Youth depression screening with parent and self-reports: assessing current and prospective depression risk. Child Psychiatry Hum Dev.

[54] Coyne JC, Thompson R, Racioppo MW (2001). Validity and efficiency of screening for history of depression by self-report. Psychol Assess.

[55] Cuijpers P, de Beurs DP, van Spijker BA, Berking M, Andersson G, Kerkhof AJ (2013). The effects of psychotherapy for adult depression on suicidality and hopelessness: a systematic review and meta-analysis. J Affective Disord.

[56] Beck AT, Weissman A, Lester D, Trexler L (1974). The measurement of pessimism: the hopelessness scale. J Consult Clin Psychol.

[57] Greenberg LS, Auszra L, Herrmann IR (2007). The relationship among emotional productivity, emotional arousal and outcome in experiential therapy of depression. Psychother Res.

[58] Oatley K, Keltner D, Jenkins J (2006). Understanding Emotions, 2nd Edition.

[59] Scheff TJ (2016). Shame and the social bond: a sociological theory. Sociol Theory.

[60] Farrand P, Perry J, Lee C, Parker M (2006). Adolescents' preference towards self-help: implications for service development. Primary Care Community Psychiatry.

[61] Uddin MZ, Dysthe KK, Følstad A, Brandtzaeg PB (2021). Deep learning for prediction of depressive symptoms in a large textual dataset. Neural Comput Applic.

[62] Burns D, Beck A (1978). Cognitive behavior modification of mood disorders. Cognitive Behavior Therapy.

[63] Brandtzæg P, Skjuve M, Dysthe K, Følstad A (2021). When the social becomes non-human: young people's perception of social support in chatbots. Proceedings of the 2021 CHI Conference on Human Factors in Computing Systems.

[64] Sanson AV, Van Hoorn J, Burke SE (2019). Responding to the impacts of the climate crisis on children and youth. Child Dev Perspect.

